# Risk Perception of Pregnancy Promotes Disapproval of Gestational Surrogacy: Analysis of a Nationally Representative Opinion Survey in Japan

**Published:** 2011-09-23

**Authors:** Kohta Suzuki, Rintaro Sawa, Kaori Muto, Satoshi Kusuda, Kouji Banno, Zentaro Yamagata

**Affiliations:** 1 Department of Health Sciences, School of Medicine, University of Yamanashi, Yamanashi, Japan; 2 Department of Obstetrics and Gynecology, Nippon Medical School, Tokyo, Japan; 3 Japan Medical Association Research Institute, Tokyo, Japan; 4 Department of Public Policy, Human Genome Center, The Institute of Medical Science, The University of Tokyo, Tokyo, Japan; 5 Maternal and Perinatal Center, Tokyo Women’s Medical University, Tokyo, Japan; 6 Department of Obstetrics and Gynecology, Keio University School of Medicine, Tokyo, Japan

**Keywords:** Risk Assessment, Surrogate Mothers, Public Opinion, Infertility, Gestational Pregnancy

## Abstract

**Background:**

To clarify the relationship between the general attitude towards gestational surrogacy and risk perception about pregnancy and infertility treatment.

**Materials and Methods:**

This study analysed the data of nationally representative cross-sectional surveys from 2007 concerning assisted reproductive technologies. The participants represented the general Japanese population. We used this data to carry out multivariate analysis. The main outcome measures were adjusted odds ratios and 95% confidence intervals from logistic regression models for factors including the effect of pregnancy risk perception on the attitude toward gestational surrogacy.

**Results:**

In this survey, 3412 participants responded (response rate of 68.2%). With regard to the attitude towards gestational surrogacy, 54.0% of the respondents approved of it, and 29.7% stated that they were undecided. The perception of a high level of risk concerning ectopic pregnancy, threatened miscarriage or premature birth, and pregnancy-induced hypertension influenced the participants’ attitudes towards gestational surrogacy. Moreover, this perception of risk also contributed to a disapproval of the technique.

**Conclusion:**

Our findings suggest that a person who understands the risks associated with pregnancy might clearly express their disapproval of gestational surrogacy.

## Introduction

Although gestational surrogacy offers several advantages, certain ethical and legal concerns regarding this procedure have arisen. In the years 1998, 2001, 2004, and 2007, the International Federation of Fertility Societies (IFFS) issued a report regarding the worldwide implementation of gestational surrogacy (1-4). The latest report in 2007 indicated that gestational surrogacy is employed in approximately one-third of all surveyed countries and regions, and that jurisdictions often have special legal requirements for the same (4).

Implementation of gestational surrogacy was restricted due to the establishment of various guidelines or legal requirements for this procedure because it included the participation of a surrogate mother who might be exposed to the risks of pregnancy. Therefore, examining the perception of pregnancy risk in the general population is important for the implementation of gestational surrogacy.

Previous studies have surveyed the association between general attitudes towards new biotechnologies in medicine that remain controversial and the risk perception of these technologies. For example, in the case of prenatal testing for Down syndrome, a significant relationship was found between the prenatal testing strategy and the perceived procedure- related miscarriage risk (5). Moreover, with regard to reproductive technology, 58% of the people who were aware of *in vitro* fertilization in Japan thought that it was a valuable procedure, although 71% were concerned about its use and 41% were either very or extremely concerned about its use (6).

However, with regard to gestational surrogacy, no studies have examined the relationship between the general attitude towards it and risk perception of pregnancy and fertility treatments.

In 2007, a third nationwide opinion survey on assisted reproductive technology (ART), including opinions on gestational surrogacy, was conducted in Japan. This survey also questioned participants regarding their perceptions of the risks associated with pregnancy and infertility treatments.

Identical nationwide opinion surveys on ARTs, including gestational surrogacy, were conducted in 1999 and 2003. In both surveys, approximately half of the respondents approved of gestational surrogacy, while 20-30% disapproved of the procedure. Our previous results suggested that older people and highly educated people with a relatively deeper knowledge of pregnancy or infertility treatments were likely to disapprove of gestational surrogacy (7).

Therefore, we hypothesized that people with a high perception of the risks associated with pregnancy or infertility treatments might be likely to disapprove of gestational surrogacy. This study aims to examine this hypothesis by using a nationally representative opinion survey.

## Materials and Methods

We analysed the data of the National Survey of People’s Attitudes towards ART involving donors and surrogate mothers, conducted by the Ministry of Health, Labour and Welfare in February and March 2007. This was a cross-sectional survey. We obtained permission from the government to use the national data.

### Outline of the 2007 survey

The 2007 survey was conducted in accordance with the 1999 and 2003 surveys. Detailed outlines of the previous surveys have been provided by Suzuki et al. (7). Briefly, the participants were selected using a stratified two-step randomization procedure to ensure that they represented the general Japanese population. The questionnaire was completed anonymously.

### Primary outcomes

We used the data of the above survey concerning the attitudes towards gestational surrogacy. The answers to the following questions and responses were analysed as dependent variables.

‘If the situation arose, would you consider using gestational surrogacy?’

1. I would consider using gestational surrogacy.
2. I would consider using this technique only if my partner agrees to it.
3. I do not want to use gestational surrogacy.

‘In general, are you of the opinion that the use of gestational surrogacy by couples in whom the condition of the wife’s womb prevents pregnancy should be approved by society?’

1. Should be approved.
2. Should not be approved.
3. I am undecided.

#### 1. Evaluation of the effect of the risk perception of pregnancy and infertility treatments on the attitude towards personal use of gestational surrogacy

In order to identify the effect of the risk perception of pregnancy and infertility treatments on the attitude towards personal use of gestational surrogacy, multivariate analyses were performed using ‘I would consider using gestational surrogacy’ and ‘I do not want to use gestational surrogacy’ (the answers were ‘I would consider using this technique only if my partner agrees to it’ and ‘I do not want to use gestational surrogacy,’ respectively) as dependent variables.

#### 2. Evaluation of the effect of risk perception of pregnancy and infertility treatments on an individual’s ability to clearly express his/her opinion on gestational surrogacy

When discussing the general attitude towards gestational surrogacy, an individual’s ability to express his/her opinion is considered to be important. In order to identify the factors that affect this ability, multivariate (multiple logistic) analysis was performed with regard to the effects of the independent variables. This analysis was performed using the responses ‘I can decide’ (the answers were either ‘Should be approved’ or ‘Should not be approved’) and ‘I am undecided’ (the answer was ‘I cannot decide’) as dependent variables.

#### 3. Evaluation of the effect of risk perception of pregnancy and infertility treatments on the pros and cons of gestational surrogacy

If people are able to express their opinions regarding gestational surrogacy, these opinions could be used in drafting laws and preparing guidelines pertaining to gestational surrogacy. In order to identify the factors that greatly affect the general attitude towards gestational surrogacy, multivariate analyses were performed using ‘Should be approved’ and ‘Should not be approved’ as dependent variables.

### Independent variables

 Demographic and socioeconomic variables were based on certain national surveys in Japan (e.g., the Comprehensive Survey of Living Conditions of the People on Health and Welfare). Details of these variables have been described by Suzuki et al. (7). In this study, we added the variables about risk perception of pregnancy and infertility treatment.

The answers to eight questions regarding this risk perception were analysed. The validity of these questions was not examined, and it might be difficult to analyse these questions as a whole. Therefore, factor analysis was initially performed, and the eight questions were classified on the basis of the risk perception of three groups of factors: ectopic pregnancy, threatened miscarriage or premature birth, and pregnancy-induced hypertension (pregnancy risk 1); premature rupture of membranes and abnormality of the placenta (pregnancy risk 2); and infertility treatments (treatment risk). In each group, internal coherence was acceptable with a Cronbach’s α coefficient over 0.75.

These questions were answered on a scale of 1 to 3, where 1 indicated ‘well known,’ 2 indicated ‘little known,’ and 3 indicated ‘unknown’. The scores of these answers were totalled, and this total score was analysed using quantile regression techniques.

### Statistical analyses

In order to estimate the effect of gender, age class, socioeconomic background, and risk perception on the attitude towards gestational surrogacy, two models were designed, and multivariable analysis using multiple logistic analyses was performed to evaluate the effects of the independent variables.

Model 1: To compare the results of our past and present studies, we used only gender, age class, and socioeconomic factors as independent variables.
Model 2: To examine the effect of the risk perception of pregnancy and infertility treatments on the general attitude towards gestational surrogacy considering socioeconomic backgrounds, we used the variables of model 1 and risk perception of pregnancy and infertility treatments as independent variables.

Statistical analyses were performed using the statistical software SAS version 9.1.3 (SAS Institute, Inc., Cary, North Carolina, USA). Significance was set at p < 0.05 in all statistical analyses.

## Results

### Survey

**Fig 1:**
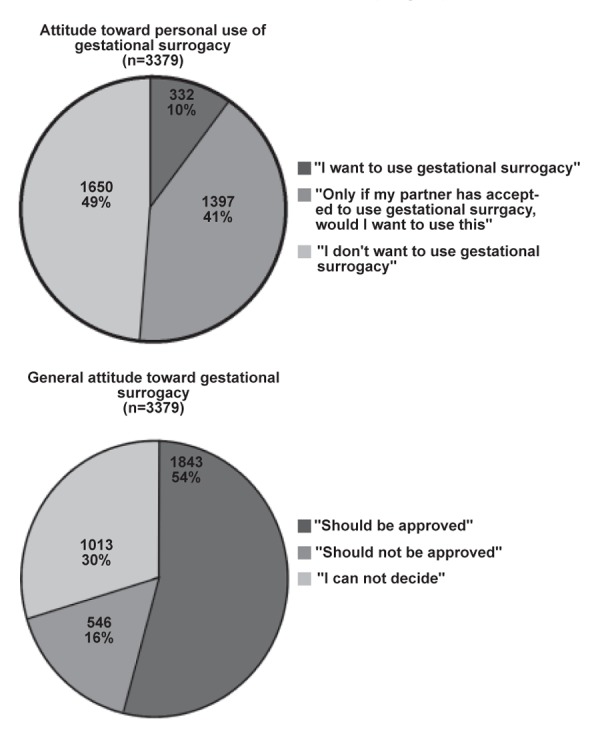
Distribution of responses to the following questions: ‘If you were infertile, would you consider using gestational surrogacy?’ and ‘In general, do you consider that the use of gestational surrogacy by couples in whom the condition of the wife’s womb prevents pregnancy should be approved by society?’

In the present survey, 5000 participants received the questionnaire, and 3412 responded (68.2%). Among the respondents, 332 people (9.7%) stated that they would consider using gestational surrogacy, and 1397 people (40.9%) answered that they would consider using it only if their partners agreed to it. With regard to their attitude towards gestational surrogacy, 1843 (54.0%) of the respondents approved of it, and 1013 (29.7%) stated that they were undecided on the matter (Fig 1).

The mean score of pregnancy risk 1 was 4.1 (range 3-9); that of pregnancy risk 2 was 3.3 (range 2-6); and that of the treatment risk was 7.1 (range 3-9).

1. Evaluation of the effect of the risk perception of pregnancy and infertility treatments on the attitude towards personal use of gestational surrogacy (Table 1).
  In model 1, people over 40 years of age, married people, people who were childless, and people in professional occupations were unlikely to choose the response ‘I would consider using gestational surrogacy.’
  In model 2, we estimated the effect of the risk perception of pregnancy and infertility treatments on the attitude towards personal use of gestational surrogacy. However, in this model, there were no effects of risk perception. With regard to gender, age group, and socioeconomic factors, we found the results to be similar to those of model 1.
2. Evaluation of the effect of the risk perception of pregnancy and infertility treatments on an individual’s ability to clearly express his/her opinion on gestational surrogacy (Table 2).
  In model 1, factors related to the inability to express a clear opinion included age over 60 years. Professionals and highly educated people clearly expressed their opinions on this issue. People with the highest household incomes and those in the lower middle class also clearly expressed their opinions.
  In model 2, with regard to gender, females were more likely to be unable to clearly express their opinions than males. With regard to other socioeconomic factors, we found the results to be similar to those for model 1. Moreover, with regard to risk perception, people who perceived a high level of pregnancy risk 1 were more likely to express their opinions.
3. Evaluation of the effect of the risk perception of pregnancy and infertility treatments on the pros and cons of gestational surrogacy (Table 3).
  In model 1, people over 30 years of age and those who had children tended to disapprove of gestational surrogacy; no other factor had an effect.
  In model 2, the perception of a high level of pregnancy risk 1 contributed to the disapproval of this technique. Further, with regard to gender, age group, and socioeconomic factors, we found the results to be almost similar to those obtained for model 1.

**Table 1:** Multivariate-adjust odds ratio (OR) and 95% confidence interval (CI) from logistic regression models for the factors that determine personal use of gestational surrogacy


	n=3379		Model 1*			Model 2**		
	I want to use	I do not want to use	OR^§^	95%	CI^§^	OR	95%	CI

**Gender**								
**Male**	114	1158	1.00			1.00		
**Female**	213	1846	1.19	0.90-	1.57	1.16	0.87-	1.55
**Age group (years)**								
**20-29**	62	430	1.00			1.00		
**30-39**	96	659	0.96	0.65-	1.41	0.95	0.65-	1.40
**40-49**	56	586	0.60	0.39-	0.94	0.61	0.39-	0.95
**50-59**	65	703	0.58	0.38-	0.90	0.57	0.37-	0.89
**60-69**	45	608	0.53	0.33-	0.85	0.54	0.33-	0.87
**Marital status**								
**Unmarried**	67	510	1.00			1.00		
**Married**	258	2487	0.50	0.25-	0.99	0.49	0.25-	0.97
**Do you have a child (ren)?**								
**Yes**	248	2310	1.00			1.00		
**No**	78	689	0.45	0.24-	0.85	0.44	0.24-	0.83
**Occupation**								
**Blue-collar worker**	45	357	1.00			1.00		
**White-collar worker Professional**	145	1156	0.88	0.61-	1.28	0.90	0.61-	1.31
**Professional**	49	553	0.64	0.41-	0.99	0.64	0.41-	1.00
**Unemployed**	88	942	0.67	0.44-	1.01	0.67	0.44-	1.02
**Annual Income (Million Yen)**								
**0.00-2.99**	63	654	1.00			1.00		
**3.00-4.99**	92	824	1.20	0.86-	1.69	1.22	0.87-	1.72
**5.00-6.99**	69	650	1.16	0.80-	1.68	1.18	0.82-	7.71
**>7.00**	99	815	1.37	0.97-	1.94	1.38	0.97-	1.95
**Education**								
**Junior high school **	19	204	1.00			1.00		
**High school**	135	1308	1.06	0.64-	1.76	1.04	0.63-	1.73
**Occupational school or junior college**	104	809	1.22	0.72-	2.07	1.21	0.71-	2.05
**University or graduate school**	71	688	1.08	0.621-	1.88	1.06	0.61-	1.85
**Risk perception of ectopic pregnancy, threatened premature miscarriage, or threatened premature birth, and pregnancy induced hypertension.**								
**Low risk perception**	155	1502				1.00		
**High risk perception**	177	1531				1.10	0.82-	1.46
**Risk perception of premature rupture of membranes and abnormality of the plancnt. **								
**Low risk perception**	135	1307				1.00		
**High risk perception**	197	1729				0.89	0.66-	1.20
**Risk perception of infertility treatment**								
**Low risk perception**	156	1558				1.00		
**High risk perception**	175	1466				1.16	0.90-	1.48


* Adjust for gender, age and socioeconomic factors ** Adjust for gender, age, socioeconomic factors and risk perception § OR, odds ratio; CI, confidence interval

**Table 2:** Multivariate-adjust odds ratio (OR) and 95% confidence interval (CI) from logistic regression models for the factors that influence a person to have a clear opinion on gestational surrogacy


	n=3402		Model 1*			Model 2**		
	I want to use	I do not want to use	OR^§^	95%	CI^§^	OR	95%	CI

**Gender**								
**Male**	928	349	1.00			1.00		
**Female**	1426	651	0.92	0.77-	1.10	0.81	0.68-	0.98
**Age group (years)**								
**20-29**	370	125	1.00			1.00		
**30-39**	562	195	0.97	0.73-	1.29	0.96	0.72-	1.28
**40-49**	462	187	0.79	0.58-	1.07	0.76	0.56-	1.03
**50-59**	543	233	0.78	0.58-	1.05	0.79	0.58-	1.07
**60-69**	410	247	0.62	0.46-	0.84	0.65	0.47-	0.89
**Marital status**								
**Unmarried**	418	162	1.00			1.00		
**Married**	1930	834	1.21	0.82-	1.77	1.12	0.76-	1.64
**Do you have a child (ren)?**								
**Yes**	1790	788	1.00			1.00		
**No**	559	211	1.12	0.80-	1.55	1.21	0.86-	1.68
**Occupation**								
**Blue-collar worker**	281	125	1.00			1.00		
**White-collar worker**	906	401	0.98	0.76-	1.25	0.99	0.77-	1.27
**Professional**	476	130	1.45	1.08-	1.94	1.47	1.09-	1.98
**Unemployed**	701	336	1.03	0.79-	1.34	1.03	0.79-	1.35
**Annual Income (Million Yen)**								
**0.00-2.99**	473	247	1.00			1.00		
**3.00-4.99**	667	260	1.32	1.07-	1.63	1.33	1.07-	1.65
**5.00-6.99**	506	217	1.12	0.89-	1.41	1.11	0.88-	1.40
**>7.00**	682	235	1.33	1.06-	1.67	1.29	1.02-	1.61
**Education**								
**Junior high school **	137	88	1.00			1.00		
**High school**	975	478	1.16	0.87-	1.54	1.15	0.87-	1.54
**Occupational school or junior college**	662	259	1.31	0.96-	1.78	1.28	0.94-	1.75
**University or graduate school**	591	171	1.63	1.17-	2.26	1.58	1.13-	2.20
**Risk perception of ectopic pregnancy, threatened premature miscarriage, or threatened premature birth, and pregnancy induced hypertension.**								
**Low risk perception**	1121	547				1.00		
**High risk perception**	1261	459				1.28	1.6-	1.54
**Risk perception of premature rupture of membranes and abnormality of the plancnt. **								
**Low risk perception**	974	476				1.00		
**High risk perception**	1409	532				1.18	0.98-	1.43
**Risk perception of infertility treatment**								
**Low risk perception**	1170	556				1.00		
**High risk perception**	1202	449				1.10	0.93-	1.30


* Adjust for gender, age and socioeconomic factors ** Adjust for gender, age, socioeconomic factors and risk perception § OR, odds ratio; CI, confidence interval

**Table 3:** Multivariate-adjust odds ratio (OR) and 95% confidence interval (CI) from logistic regression models for the factors that influence people,s opinion of the attitude towards gestational


	n=2389		Model 1*			Model 2**		
	I want to use	I do not want to use	OR^§^	95%	CI^§^	OR	95%	CI

**Gender**								
**Male**	701	227	1.00			1.00		
**Female**	1114	312	1.07	0.85-	1.35	1.12	0.88-	1.43
**Age group (years)**								
**20-29**	327	43	1.00			1.00		
**30-39**	475	87	0.64	0.42-	0.98	0.66	0.43-	1.02
**40-49**	362	100	0.41	0.26-	0.63	0.42	0.27-	0.65
**50-59**	377	166	0.25	0.16-	0.38	0.25	0.16-	0.39
**60-69**	267	143	0.22	0.14-	0.34	0.22	0.14-	0.35
**Marital status**								
**Unmarried**	351	67	1.00			1.00		
**Married**	1458	472	0.68	0.43-	1.10	0.69	0.43-	1.10
**Do you have a child (ren)?**								
**Yes**	1362	428	1.00			1.00		
**No**	448	111	0.58	0.40-	0.85	0.57	0.39-	0.84
**Occupation**								
**Blue-collar worker**	227	54	1.00			1.00		
**White-collar worker**	718	188	0.90	0.63-	1.27	0.93	0.65-	1.31
**Professional**	356	120	0.72	0.50-	1.04	0.75	0.52-	1.09
**Unemployed**	524	177	0.78	0.54-	1.13	0.80	0.555-	1.17
**Annual Income (Million Yen)**								
**0.00-2.99**	362	111	1.00			1.00		
**3.00-4.99**	520	147	1.18	0.88-	1.57	1.21	0.90-	1.61
**5.00-6.99**	383	123	1.09	0.80-	1.49	1.12	0.82-	1.53
**>7.00**	534	148	1.29	0.95-	1.75	1.35	0.99-	1.83
**Education**								
**Junior high school **	102	35	1.00			1.00		
**High school**	745	230	0.94	0.63-	1.40	0.97	0.65-	1.46
**Occupational school or junior college**	533	129	1.08	0.70-	1.66	1.11	0.72-	1.71
**University or graduate school**	448	143	0.89	0.57-	1.37	0.92	0.59-	1.43
**Risk perception of ectopic pregnancy, threatened premature miscarriage, or threatened premature birth, and pregnancy induced hypertension.**								
**Low risk perception**	884	237				1.00		
**High risk perception**	953	308				0.78	0.61-	0.99
**Risk perception of premature rupture of membranes and abnormality of the plancnt. **								
**Low risk perception**	744	230				1.00		
**High risk perception**	1093	316				1.07	0.84-	1.38
**Risk perception of infertility treatment**								
**Low risk perception**	898	272				1.00		
**High risk perception**	930	272				1.02	0.82-	1.26


* Adjust for gender, age and socioeconomic factors ** Adjust for gender, age, socioeconomic factors and risk perception § OR, odds ratio; CI, confidence interval

## Discussion

In this study, for the first time, we surveyed the risk perception of pregnancy or infertility treatments in order to examine the relationship between the general attitude towards gestational surrogacy and these risk perceptions. A high level of risk perception with regard to ectopic pregnancy, threatened miscarriage or premature birth, and pregnancy-induced hypertension (pregnancy risk 1) influenced the respondents’ attitudes towards gestational surrogacy. Moreover, this perception of risk also contributed to their disapproval of this technique.

We considered that pregnancy risk 1 was a more serious threat to mothers and, hence, it was more important to prevent pregnancy risk 1 than premature rupture of membranes and abnormality of the placenta (pregnancy risk 2) (8-11). The procedure of gestational surrogacy involves the pregnancy of a surrogate mother. The surrogate mother might therefore be exposed to the risks associated with pregnancy. It is necessary for surrogate mothers to avoid the complications associated with pregnancy. Therefore, they must understand the risks of pregnancy.

In international debates regarding surrogacy, it is opined that the female reproductive function tends to be regarded as a commodity in commercial surrogacy, which leads to the exploitation of women (12, 13). The possibility of the participation of socially handicapped people in surrogacy for compensation alone, with insufficient understanding of the risks involved, has been suggested. In a previous study, we discussed the necessity of informing socially handicapped people about the various aspects of surrogacy (7). Our present results also support this necessity, particularly with regard to the perception of pregnancy risk.

On the other hand, no relationship was found between pregnancy risk 2 and attitudes towards surrogacy. With regard to the premature rupture of membranes and abnormality of the placenta, there is a relatively low risk for the mother (8-11).

There were few differences between model 1 and model 2 with respect to the ORs for other factors, including socioeconomic factors. Our results suggest that risk perception is an independent factor that affects people’s attitudes. In a previous study concerning prenatal testing for Down syndrome, it was described how racial, ethnic, and other socioeconomic differences in the prenatal testing strategy were mediated by risk perception (5). Our results are consistent with this study with regard to the relationship between socioeconomic factors and risk perception.

For the first time, we succeeded in clarifying the factors associated with attitudes towards personal use of gestational surrogacy. A person who is married or childless was unlikely to use gestational surrogacy. These results are similar to the results regarding the pros and cons of surrogacy. However, in the 2003 survey, neither marriage nor children were associated with the general attitude towards surrogacy (7). These differences suggest that between 2003 and 2007 married people or parents in Japan might have changed their attitudes and begun to disapprove of surrogacy.

The present study does, however, have certain limitations. We were unable to clarify a causal relationship between the factors and attitudes towards gestational surrogacy because this study was only cross-sectional in nature. However, the causal relationship may not be important because it might be difficult to change public opinion. Moreover, we used the data of three surveys to clarify the trend in people’s attitudes and the changes in the factors that are associated with the general attitude from 1999 to 2007. When we discuss these issues, it may be important to grasp the changing trend in attitude. In this respect, our results might contribute to future discussions on gestational surrogacy. Despite their limitation, our findings could be considered to be representative of the current general attitude towards gestational surrogacy since this study was based on a nationwide opinion survey.

## Conclusion

Our findings suggest that the perception of a high level of risk concerning ectopic pregnancy, threatened miscarriage or premature birth, and pregnancy- induced hypertension might influence people’s attitudes towards gestational surrogacy. Moreover, this perception of risk might contribute to their disapproval of the technique. These results suggest that a person who perceives high risks to be associated with pregnancy might express his/her clear disapproval of gestational surrogacy. It is essential to consider the perception of pregnancy risk in further discussions on this topic.
